# Supplementary material for: Genetic Diversity and Population Structure of the Asian Tiger Mosquito (Aedes albopictus) in Vietnam: Evidence for Genetic Differentiation by Climate Region
Source: Genes (Basel). 2021 Oct 6;12(10):1579. doi: 10.3390/genes12101579 (PMC8535633; doi:10.3390/genes12101579)
Supplement: Supplementary file 1 [file genes-12-01579-s001.zip › Supp Table S1. Collection sites.pdf]

Table S1. Detailed information on the 236 individual *Aedes albopictus* samples from 16 localities in Vietnam

No	Sample Code	Locality	Latitude	Longitude	Elevation (m)	Collection date	Collected Stage	Habitat	Analyzed stage	Sex	Collectors
1	LC01	Lai Chau city, Lai Chau	22.394021	103.462878	910	3/7/2019	Larvae	Tire	Adult	Male	C.V.D. and V.V.N
2	LC02	Lai Chau city, Lai Chau	22.393654	103.462749	910	3/7/2019	Larvae	Water container	Adult	Male	C.V.D. and V.V.N
3	LC03	Lai Chau city, Lai Chau	22.388932	103.463382	912	3/7/2019	Larvae	Discarded trash pot	Adult	Female	C.V.D. and V.V.N
4	LC04	Lai Chau city, Lai Chau	22.38825	103.46001	959	3/7/2019	Larvae	Bamboo stump	Adult	Female	C.V.D. and V.V.N
5	LC05	Lai Chau city, Lai Chau	22.394993	103.46012	911	3/7/2019	Adult	Resident garden	Adult	Male	C.V.D. and V.V.N
6	LC06	Lai Chau city, Lai Chau	22.395489	103.457116	916	3/7/2019	Adult	Resident garden	Adult	Female	C.V.D. and V.V.N
7	LC07	Lai Chau city, Lai Chau	22.399546	103.457738	923	3/7/2019	Larvae	Park-Water container	Adult	Female	C.V.D. and V.V.N
8	LC08	Lai Chau city, Lai Chau	22.396759	103.454037	931	3/7/2019	Larvae	Tire	Adult	Female	C.V.D. and V.V.N
9	LC09	Lai Chau city, Lai Chau	22.396392	103.44892	931	3/7/2019	Adult	Grassland	Adult	Male	C.V.D. and V.V.N
10	LC10	Lai Chau city, Lai Chau	22.399792	103.443008	922	3/7/2019	Adult	Grassland	Adult	Male	C.V.D. and V.V.N
11	LC10	Lai Chau city, Lai Chau	22.38825	103.46001	959	3/7/2019	Larvae	Bamboo stump	Adult	Female	C.V.D. and V.V.N
12	LC10	Lai Chau city, Lai Chau	22.38825	103.46001	959	3/7/2019	Larvae	Bamboo stump	Adult	Female	C.V.D. and V.V.N
13	LC10	Lai Chau city, Lai Chau	22.399546	103.457738	923	3/7/2019	Adult	Park-scrub	Adult	Female	C.V.D. and V.V.N
14	LC10	Lai Chau city, Lai Chau	22.399546	103.457738	923	3/7/2019	Adult	Park-scrub	Adult	Female	C.V.D. and V.V.N
15	LC10	Lai Chau city, Lai Chau	22.399543	103.457723	923	3/7/2019	Adult	Park-scrub	Adult	Female	C.V.D. and V.V.N
16	DB01	Dien Bien Phu city, Dien	21.393013	103.024629	487	5/7/2019	Larvae	Tire	Adult	Male	C.V.D. and V.V.N
17	DB02	Dien Bien Phu city, Dien	21.394779	103.0206	492	5/7/2019	Larvae	Tire	Adult	Male	C.V.D. and V.V.N
18	DB03	Dien Bien Phu city, Dien	21.396367	103.015611	495	5/7/2019	Larvae	Plastic container	Adult	Male	C.V.D. and V.V.N
19	DB04	Dien Bien Phu city, Dien	21.390833	103.014753	484	5/7/2019	Larvae	Cement tank	Adult	Female	C.V.D. and V.V.N
20	DB05	Dien Bien Phu city, Dien	21.387756	103.013069	477	5/7/2019	Larvae	Flower pot	Adult	Female	C.V.D. and V.V.N
21	DB06	Dien Bien Phu city, Dien	21.3833	103.014196	482	5/7/2019	Larvae	Bonsai bucket	Adult	Male	C.V.D. and V.V.N
22	DB07	Dien Bien Phu city, Dien	21.398819	103.009641	483	5/7/2019	Larvae	Tire	Adult	Female	C.V.D. and V.V.N
23	DB08	Dien Bien Phu city, Dien	21.394918	103.010596	483	5/7/2019	Larvae	Bamboo stump	Adult	Female	C.V.D. and V.V.N
24	DB09	Dien Bien Phu city, Dien	21.407305	103.002281	485	5/7/2019	Adult	Park-scrub	Adult	Female	C.V.D. and V.V.N
25	DB10	Dien Bien Phu city, Dien	21.409023	102.994846	499	5/7/2019	Larvae	Park-flower pot	Adult	Female	C.V.D. and V.V.N
26	DB11	Dien Bien Phu city, Dien	21.398819	103.009641	483	5/7/2019	Larvae	Pet's food container	Adult	Male	C.V.D. and V.V.N
27	DB12	Dien Bien Phu city, Dien	21.398819	103.009641	483	5/7/2019	Larvae	Pet's food container	Adult	Female	C.V.D. and V.V.N
28	DB13	Dien Bien Phu city, Dien	21.409023	102.994846	499	5/7/2019	Larvae	Rock hole	Adult	Male	C.V.D. and V.V.N
29	DB14	Dien Bien Phu city, Dien	21.409023	102.994846	499	5/7/2019	Larvae	Cement tank	Adult	Female	C.V.D. and V.V.N
30	DB15	Dien Bien Phu city, Dien	21.394918	103.010596	483	5/7/2019	Larvae	Bamboo stump	Adult	Female	C.V.D. and V.V.N
31	SL01	Son La city, Son La prov	21.29897	103.91367	677	7/7/2019	Larvae	Flower pot	Adult	Male	C.V.D. and V.V.N
32	SL02	Son La city, Son La prov	21.325846	103.916194	615	7/7/2019	Larvae	Cement tank	Adult	Male	C.V.D. and V.V.N
33	SL03	Son La city, Son La prov	21.321693	103.918957	606	7/7/2019	Larvae	Bonsai bucket	Adult	Female	C.V.D. and V.V.N
34	SL04	Son La city, Son La prov	21.319594	103.919933	619	7/7/2019	Larvae	Bamboo stump	Adult	Female	C.V.D. and V.V.N
35	SL05	Son La city, Son La prov	21.316336	103.918066	622	7/7/2019	Adult	Resident garden	Adult	Female	C.V.D. and V.V.N
36	SL06	Son La city, Son La prov	21.317186	103.913034	630	7/7/2019	Adult	Resident garden	Adult	Male	C.V.D. and V.V.N
37	SL07	Son La city, Son La prov	21.313628	103.922819	620	7/7/2019	Larvae	Tire	Adult	Male	C.V.D. and V.V.N
38	SL08	Son La city, Son La prov	21.318725	103.930753	686	7/7/2019	Adult	Bamboo forest	Adult	Male	C.V.D. and V.V.N
39	SL09	Son La city, Son La prov	21.311334	103.930517	639	7/7/2019	Adult	Park	Adult	Female	C.V.D. and V.V.N
40	SL10	Son La city, Son La prov	21.301433	103.929546	655	7/7/2019	Adult	Park	Adult	Female	C.V.D. and V.V.N
41	SL11	Son La city, Son La prov	21.318725	103.930753	686	7/7/2019	Adult	Resident garden	Adult	Female	C.V.D. and V.V.N
42	SL12	Son La city, Son La prov	21.318725	103.930753	686	7/7/2019	Adult	Resident garden	Adult	Female	C.V.D. and V.V.N
43	SL13	Son La city, Son La prov	21.318725	103.930753	686	7/7/2019	Adult	Bamboo forest	Adult	Female	C.V.D. and V.V.N
44	SL14	Son La city, Son La prov	21.318725	103.930753	686	7/7/2019	Adult	Bamboo forest	Adult	Female	C.V.D. and V.V.N
45	SL15	Son La city, Son La prov	21.318725	103.930753	686	7/7/2019	Adult	Bamboo forest	Adult	Female	C.V.D. and V.V.N
46	HG01	Ha Giang city, Ha Giang	22.828534	104.985832	106	19/07/2019	Adult	Play ground-plastic container	Adult	Female	C.V.D. and V.V.N
47	HG02	Ha Giang city, Ha Giang	22.827298	104.990242	129	19/07/2019	Adult	Resident garden	Adult	Female	C.V.D. and V.V.N
48	HG03	Ha Giang city, Ha Giang	22.833236	104.985382	104	19/07/2019	Larvae	Bamboo stump	Adult	Female	C.V.D. and V.V.N
49	HG04	Ha Giang city, Ha Giang	22.834957	104.991562	105	19/07/2019	Larvae	Ground pool water	Adult	Female	C.V.D. and V.V.N
50	HG05	Ha Giang city, Ha Giang	22.822668	104.987826	106	19/07/2019	Larvae	Tire	Adult	Male	C.V.D. and V.V.N
51	HG06	Ha Giang city, Ha Giang	22.817622	104.988566	105	19/07/2019	Larvae	Tire	Adult	Male	C.V.D. and V.V.N
52	HG07	Ha Giang city, Ha Giang	22.825869	104.98347	109	19/07/2019	Larvae	Coconut	Adult	Female	C.V.D. and V.V.N
53	HG08	Ha Giang city, Ha Giang	22.819738	104.981517	103	19/07/2019	Larvae	Tire	Adult	Female	C.V.D. and V.V.N
54	HG09	Ha Giang city, Ha Giang	22.819892	104.987194	108	19/07/2019	Adult	Resident garden	Adult	Female	C.V.D. and V.V.N
55	HG10	Ha Giang city, Ha Giang	22.813899	104.988696	109	19/07/2019	Adult	Resident garden	Adult	Female	C.V.D. and V.V.N
56	HG11	Ha Giang city, Ha Giang	22.814899	104.983232	108	19/07/2020	Adult	Forest (Arboretum)	Adult	Male	C.V.D. and V.V.N
57	HG12	Ha Giang city, Ha Giang	22.814899	104.983232	108	19/07/2021	Adult	Forest (Arboretum)	Adult	Male	C.V.D. and V.V.N
58	HG13	Ha Giang city, Ha Giang	22.814899	104.983232	108	19/07/2022	Adult	Forest (Arboretum)	Adult	Male	C.V.D. and V.V.N
59	HG14	Ha Giang city, Ha Giang	22.814899	104.983232	108	19/07/2023	Adult	Resident garden	Adult	Female	C.V.D. and V.V.N
60	HG15	Ha Giang city, Ha Giang	22.833804	104.985431	114	19/07/2024	Larvae	Park-Water container	Adult	Female	C.V.D. and V.V.N
61	HG16	Ha Giang city, Ha Giang	22.833804	104.985431	114	19/07/2025	Adult	Park-scrub	Adult	Female	C.V.D. and V.V.N
62	HG17	Ha Giang city, Ha Giang	22.833804	104.985431	114	19/07/2026	Adult	Park-scrub	Adult	Female	C.V.D. and V.V.N
63	CB01	Cao Bang city, Cao Bang	22.670478	106.25514	188	23/07/2019	Adult	Brust	Adult	Male	C.V.D. and V.V.N
64	CB02	Cao Bang city, Cao Bang	22.672389	106.256095	189	23/07/2019	Adult	Grassland	Adult	Male	C.V.D. and V.V.N
65	CB03	Cao Bang city, Cao Bang	22.669063	106.25794	192	23/07/2019	Adult	Grassland	Adult	Female	C.V.D. and V.V.N
66	CB04	Cao Bang city, Cao Bang	22.665865	106.257135	194	23/07/2019	Adult	Forest (Arboretum)	Adult	Female	C.V.D. and V.V.N
67	CB05	Cao Bang city, Cao Bang	22.661806	106.258691	195	23/07/2019	Adult	Forest (Arboretum)	Adult	Female	C.V.D. and V.V.N
68	CB06	Cao Bang city, Cao Bang	22.664004	106.260204	193	23/07/2019	Adult	Park-Water container	Adult	Female	C.V.D. and V.V.N
69	CB07	Cao Bang city, Cao Bang	22.66544	106.266491	212	23/07/2019	Adult	Park-Water container	Adult	Female	C.V.D. and V.V.N
70	CB08	Cao Bang city, Cao Bang	22.669836	106.266437	198	23/07/2019	Adult	Grassland	Adult	Female	C.V.D. and V.V.N
71	CB09	Cao Bang city, Cao Bang	22.668638	106.262843	193	23/07/2019	Adult	Tire	Adult	Female	C.V.D. and V.V.N
72	CB10	Cao Bang city, Cao Bang	22.673806	106.260354	204	23/07/2019	Adult	Tire	Adult	Female	C.V.D. and V.V.N
73	CB11	Cao Bang city, Cao Bang	22.669836	106.266437	198	23/07/2020	Adult	Grassland	Adult	Female	C.V.D. and V.V.N
74	CB12	Cao Bang city, Cao Bang	22.66544	106.266491	212	23/07/2021	Adult	Park-scrub	Adult	Female	C.V.D. and V.V.N
75	CB13	Cao Bang city, Cao Bang	22.673806	106.260354	204	23/07/2022	Adult	Tire	Adult	Female	C.V.D. and V.V.N
76	CB14	Cao Bang city, Cao Bang	22.661806	106.258691	195	23/07/2023	Adult	Forest (Arboretum)	Adult	Female	C.V.D. and V.V.N
77	CB15	Cao Bang city, Cao Bang	22.669063	106.25794	192	23/07/2024	Adult	Grassland	Adult	Female	C.V.D. and V.V.N
78	LS01	Lang Son city, Lang Son	21.84563	106.76363	264	21/07/2019	Adult	Bamboo forest	Adult	Female	C.V.D. and V.V.N
79	LS02	Lang Son city, Lang Son	21.85039	106.762316	265	21/07/2019	Adult	Bamboo forest	Adult	Female	C.V.D. and V.V.N
80	LS03	Lang Son city, Lang Son	21.850968	106.767047	261	21/07/2019	Larvae	Tire	Adult	Male	C.V.D. and V.V.N
81	LS04	Lang Son city, Lang Son	21.849972	106.772454	266	21/07/2019	Larvae	Tire	Adult	Male	C.V.D. and V.V.N
82	LS05	Lang Son city, Lang Son	21.845586	106.777153	271	21/07/2019	Larvae	Bamboo stump	Adult	Female	C.V.D. and V.V.N
83	LS06	Lang Son city, Lang Son	21.840437	106.7799	287	21/07/2019	Larvae	Bamboo stump	Adult	Male	C.V.D. and V.V.N
84	LS07	Lang Son city, Lang Son	21.842877	106.758571	269	21/07/2019	Larvae	Flower pot	Adult	Male	C.V.D. and V.V.N
85	LS08	Lang Son city, Lang Son	21.840029	106.753657	261	21/07/2019	Larvae	Flower pot	Adult	Female	C.V.D. and V.V.N
86	LS09	Lang Son city, Lang Son	21.845785	106.755132	265	21/07/2019	Larvae	Bonsai bucket	Adult	Female	C.V.D. and V.V.N
87	LS10	Lang Son city, Lang Son	21.854371	106.756583	263	21/07/2019	Adult	Tire	Adult	Male	C.V.D. and V.V.N
88	LS11	Lang Son city, Lang Son	21.854371	106.756583	263	21/07/2020	Adult	Tire	Adult	Male	C.V.D. and V.V.N
89	LS12	Lang Son city, Lang Son	21.845586	106.777153	271	21/07/2021	Adult	Bamboo stump	Adult	Female	C.V.D. and V.V.N
90	LS13	Lang Son city, Lang Son	21.85039	106.762316	265	21/07/2022	Adult	Bamboo forest	Adult	Female	C.V.D. and V.V.N
91	LS14	Lang Son city, Lang Son	21.85039	106.762316	265	21/07/2023	Adult	Bamboo forest	Adult	Female	C.V.D. and V.V.N
92	LS15	Lang Son city, Lang Son	21.840029	106.753657	261	21/07/2024	Adult	Flower pot	Adult	Female	C.V.D. and V.V.N
93	HN01	My Dinh district, Hanoi	21.01597	105.77438	10	22/6/2019	Larvae	Plastic container	Adult	Male	C.V.D. and V.V.N
94	HN02	My Dinh district, Hanoi	21.016609	105.772521	12	22/6/2019	Larvae	Cement tank	Adult	Male	C.V.D. and V.V.N
95	HN03	My Dinh district, Hanoi	21.020362	105.77211	11	22/6/2019	Larvae	Rock hole	Adult	Female	C.V.D. and V.V.N
96	HN04	My Dinh district, Hanoi	21.022625	105.773591	11	22/6/2019	Larvae	Bonsai bucket	Adult	Female	C.V.D. and V.V.N
97	HN05	My Dinh district, Hanoi	21.016736	105.775479	10	22/6/2019	Larvae	Palm tree areca	Adult	Male	C.V.D. and V.V.N
98	HN06	My Dinh district, Hanoi	21.013982	105.770415	11	22/6/2019	Larvae	Tire	Adult	Female	C.V.D. and V.V.N
99	HN07	My Dinh district, Hanoi	21.014363	105.772915	8	22/6/2019	Larvae	Tire	Adult	Female	C.V.D. and V.V.N
100	HN08	My Dinh district, Hanoi	21.025409	105.770415	9	22/6/2019	Larvae	Bonsai bucket	Adult	Male	C.V.D. and V.V.N
101	HN09	My Dinh district, Hanoi	20.995751	105.808382	18	22/6/2019	Larvae	Flower pot	Adult	Male	C.V.D. and V.V.N
102	HN10	My Dinh district, Hanoi	21.015348	105.77337	8	22/6/2019	Larvae	Tire	Adult	Female	C.V.D. and V.V.N
103	HN11	Nam Tu Liem, Hanoi	21.032092	105.764678	9	23/6/2019	Adult	Cement tank	Adult	Female	C.V.D. and V.V.N
104	HN12	Nam Tu Liem, Hanoi	20.989884	105.786993	15	23/6/2019	Adult	Resident garden	Adult	Female	C.V.D. and V.V.N
105	HN13	Bac Tu Liem, Hanoi	21.067488	105.734538	9	23/6/2019	Adult	Resident garden	Adult	Female	C.V.D. and V.V.N
106	HN14	Bac Tu Liem, Hanoi	21.067488	105.734538	9	23/6/2019	Adult	Resident garden	Adult	Female	C.V.D. and V.V.N

107	HN15	Thanh Xuan, Hanoi	20.99596	105.80801	16	23/6/2019	Adult	University campus	Adult	Female	C.V.D. and V.V.N
108	HP01	Le Chan district, Hai Pho	20.827114	106.67854	3	25/6/2019	Adult	Tire	Adult	Female	C.V.D. and V.V.N
109	HP02	Le Chan district, Hai Pho	20.825585	106.682469	3	25/6/2019	Larvae	Flower pot	Larvae	-	C.V.D. and V.V.N
110	HP03	Le Chan district, Hai Pho	20.829271	106.694486	4	25/6/2019	Adult	Tire	Adult	Female	C.V.D. and V.V.N
111	HP04	Le Chan district, Hai Pho	20.827636	106.689068	5	25/6/2019	Adult	Tire	Adult	Female	C.V.D. and V.V.N
112	HP05	Le Chan district, Hai Pho	20.825088	106.697173	4	25/6/2019	Adult	Park	Adult	Female	C.V.D. and V.V.N
113	HP06	Le Chan district, Hai Pho	20.820545	106.699072	4	25/6/2019	Larvae	Plastic container	Larvae	-	C.V.D. and V.V.N
114	HP07	Le Chan district, Hai Pho	20.816814	106.696797	4	25/6/2019	Larvae	Cement tank	Larvae	-	C.V.D. and V.V.N
115	HP08	Le Chan district, Hai Pho	20.825448	106.69272	1	25/6/2019	Adult	Grassland	Adult	Male	C.V.D. and V.V.N
116	HP09	Le Chan district, Hai Pho	20.825167	106.683697	4	25/6/2019	Adult	Plastic container	Adult	Female	C.V.D. and V.V.N
117	HP10	Le Chan district, Hai Pho	20.827293	106.679867	4	25/6/2019	Adult	Cement tank	Adult	Female	C.V.D. and V.V.N
118	HP11	Le Chan district, Hai Pho	20.827293	106.679867	4	25/6/2019	Adult	Cement tank	Adult	Female	C.V.D. and V.V.N
119	HP12	Le Chan district, Hai Pho	20.825088	106.697173	4	25/6/2019	Adult	Park-scrub	Adult	Male	C.V.D. and V.V.N
120	HP13	Le Chan district, Hai Pho	20.825088	106.697173	4	25/6/2019	Adult	Park-scrub	Adult	Male	C.V.D. and V.V.N
121	HP14	Cat Ba, Hai Phong	20.829271	106.694486	4	25/6/2019	Adult	Tire	Adult	Female	C.V.D. and V.V.N
122	HP15	Cat Ba, Hai Phong	20.827636	106.689068	5	25/6/2019	Adult	Tire	Adult	Male	C.V.D. and V.V.N
123	TH01	Hoang Hoa district, Than	19.88138	105.95152	27	8/6/2019	Adult	Bamboo forest	Adult	Female	C.V.D. and V.V.N
124	TH02	Hoang Hoa district, Than	19.883179	105.953362	16	8/6/2019	Adult	Resident garden	Adult	Female	C.V.D. and V.V.N
125	TH03	Hoang Hoa district, Than	19.882513	105.954762	13	8/6/2019	Larvae	Plastic container	Adult	Male	C.V.D. and V.V.N
126	TH04	Hoang Hoa district, Than	19.884274	105.954285	11	8/6/2019	Larvae	Plastic container	Adult	Female	C.V.D. and V.V.N
127	TH05	Hoang Hoa district, Than	19.885594	105.95432	11	8/6/2019	Larvae	Cement tank	Adult	Male	C.V.D. and V.V.N
128	TH06	Hoang Hoa district, Than	19.884934	105.95722	8	8/6/2019	Larvae	Discarded trash pot	Adult	Male	C.V.D. and V.V.N
129	TH07	Hoang Hoa district, Than	19.88687	105.958566	12	8/6/2019	Larvae	Coconut	Adult	Male	C.V.D. and V.V.N
130	TH08	Hoang Hoa district, Than	19.877608	105.952601	9	8/6/2019	Larvae	Coconut	Adult	Female	C.V.D. and V.V.N
131	TH09	Hoang Hoa district, Than	19.876438	105.950627	11	8/6/2019	Larvae	Cement tank	Adult	Female	C.V.D. and V.V.N
132	TH10	Hoang Hoa district, Than	19.878325	105.943589	116	8/6/2019	Larvae	Bonsai bucket	Adult	Female	C.V.D. and V.V.N
133	TH11	Hoang Hoa district, Than	19.881932	105.935269	11	8/6/2019	Adult	Tire	Adult	Female	C.V.D. and V.V.N
134	TH12	Hoang Hoa district, Than	19.884848	105.943015	9	8/6/2019	Adult	Cement tank	Adult	Female	C.V.D. and V.V.N
135	TH13	Hoang Hoa district, Than	19.882084	105.935891	8	8/6/2019	Adult	Bamboo forest	Adult	Male	C.V.D. and V.V.N
136	TH14	Hoang Hoa district, Than	19.884848	105.953411	15	8/6/2019	Adult	Pig farm	Adult	Female	C.V.D. and V.V.N
137	TH15	Hoang Hoa district, Than	19.884848	105.953411	15	8/6/2019	Adult	Pig farm	Adult	Female	C.V.D. and V.V.N
138	TH16	Hoang Hoa district, Than	19.877836	105.952875	10	8/6/2019	Adult	Grassland	Adult	Male	C.V.D. and V.V.N
139	TH17	Hoang Hoa district, Than	19.873457	105.949056	12	8/6/2019	Adult	Grassland	Adult	Male	C.V.D. and V.V.N
140	NA01	Quynh Luu district, Nghe	19.1431	105.67759	6	8/10/2019	Adult	Plastic container	Adult	Female	H.H.V.
141	NA02	Quynh Luu district, Nghe	19.1431	105.67759	6	8/10/2019	Adult	Plastic container	Adult	Female	H.H.V.
142	NA03	Quynh Luu district, Nghe	19.1431	105.67759	6	8/10/2019	Adult	Tire	Adult	Female	H.H.V.
143	NA04	Quynh Luu district, Nghe	19.1431	105.67759	6	8/10/2019	Adult	Tire	Adult	Male	H.H.V.
144	NA05	Quynh Luu district, Nghe	19.1431	105.67759	6	8/10/2019	Adult	Tire	Adult	Male	H.H.V.
145	NA06	Vinh city, Nghe An prov	19.1431	105.67759	6	8/10/2019	Adult	Tire	Adult	Female	H.H.V.
146	NA07	Vinh city, Nghe An prov	18.660771	105.694025	10	27/10/2019	Adult	Tire	Adult	Female	C.V.D. and V.V.N
147	NA08	Vinh city, Nghe An prov	18.657482	105.696577	12	27/10/2019	Adult	Tire	Adult	Female	C.V.D. and V.V.N
148	NA09	Vinh city, Nghe An prov	18.664696	105.703004	10	27/10/2019	Adult	Plastic container	Adult	Female	C.V.D. and V.V.N
149	NA10	Vinh city, Nghe An prov	18.667966	105.702552	9	27/10/2019	Adult	Plastic container	Adult	Female	C.V.D. and V.V.N
150	NA11	Vinh city, Nghe An prov	18.672724	105.701021	9	27/10/2019	Adult	Plastic container	Adult	Female	C.V.D. and V.V.N
151	NA12	Vinh city, Nghe An prov	18.699916	105.674676	9	27/10/2019	Adult	Plastic container	Adult	Female	C.V.D. and V.V.N
152	NA13	Vinh city, Nghe An prov	18.683967	105.685336	12	27/10/2019	Adult	Plastic container	Adult	Female	C.V.D. and V.V.N
153	NA14	Vinh city, Nghe An prov	18.651484	105.700362	9	27/10/2019	Adult	Tire	Adult	Female	C.V.D. and V.V.N
154	NA15	Vinh city, Nghe An prov	18.651007	105.701471	11	27/10/2019	Adult	Tire	Adult	Female	C.V.D. and V.V.N
155	DN01	Hoa Khanh Nam district,	16.05247	108.15341	17	23/07/2019	Adult	Plastic container	Adult	Female	H.V.H
156	DN02	Hoa Khanh Nam district,	16.05247	108.15341	17	23/07/2019	Adult	Plastic container	Adult	Female	H.V.H
157	DN03	Hoa Khanh Nam district,	16.05247	108.15341	17	23/07/2019	Adult	Plastic container	Adult	Female	H.V.H
158	DN04	Hoa Khanh Nam district,	16.05247	108.15341	17	23/07/2019	Adult	Plastic container	Adult	Female	H.V.H
159	DN05	Hoa Khanh Nam district,	16.05247	108.15341	17	23/07/2019	Adult	Tire	Adult	Male	H.V.H
160	DN06	Hoa Khanh Nam district,	16.05247	108.15341	17	23/07/2019	Adult	Tire	Adult	Male	H.V.H
161	DN07	Hoa Khanh Nam district,	16.05247	108.15341	17	23/07/2019	Adult	Tire	Adult	Female	H.V.H
162	DN08	Hoa Khanh Nam district,	16.05247	108.15341	17	23/07/2019	Adult	Resident garden	Adult	Female	H.V.H
163	DN09	Hoa Khanh Nam district,	16.05247	108.15341	17	23/07/2019	Adult	Resident garden	Adult	Female	H.V.H
164	DN10	Cam Le district, Da Nang	16.054808	108.162525	7	25/10/2019	Adult	Tire	Adult	Female	C.V.D. and V.V.N
165	DN11	Tho Quang, Son Tra, Da	16.049368	108.160305	7	25/10/2019	Adult	Tire	Adult	Female	C.V.D. and V.V.N
166	DN12	Tho Quang, Son Tra, Da	16.065317	108.186214	11	25/10/2019	Adult	Tire	Adult	Female	C.V.D. and V.V.N
167	DN13	Tho Quang, Son Tra, Da	16.097838	108.246211	8	25/10/2019	Adult	Tire	Adult	Female	C.V.D. and V.V.N
168	DN14	An Hai Bac, Son Tra, Đ	16.083334	108.237917	13	25/10/2019	Adult	Tire	Adult	Female	C.V.D. and V.V.N
169	QN01	Binh Long district, Quan	15.279138	108.76936	14	25/10/2019	Larvae	Bonsai bucket	Larvae	-	T.T.T.D
170	QN02	Binh Long district, Quan	15.279138	108.76936	14	15/09/2019	Larvae	Plastic container	Larvae	-	T.T.T.D
171	QN03	Binh Long district, Quan	15.279138	108.76936	14	15/09/2019	Larvae	Plastic container	Larvae	-	T.T.T.D
172	QN04	Binh Long district, Quan	15.279138	108.76936	14	15/09/2019	Larvae	Resident garden	Larvae	-	T.T.T.D
173	QN05	Binh Long district, Quan	15.279138	108.76936	14	15/09/2019	Larvae	Plastic container	Larvae	-	T.T.T.D
174	QN06	Binh Long district, Quan	15.279138	108.76936	14	15/09/2019	Larvae	Plastic container	Larvae	-	T.T.T.D
175	QN07	Binh Long district, Quan	15.279138	108.76936	14	15/09/2019	Larvae	Flower pot	Larvae	-	T.T.T.D
176	QN08	Binh Long district, Quan	15.279138	108.76936	14	15/09/2019	Larvae	Flower pot	Larvae	-	T.T.T.D
177	QN09	Binh Long district, Quan	15.279138	108.76936	14	15/09/2019	Larvae	Plastic container	Larvae	-	T.T.T.D
178	QN10	Binh Long district, Quan	15.279138	108.76936	14	15/09/2019	Larvae	Plastic container	Larvae	-	T.T.T.D
179	QN11	Binh Long district, Quan	15.279138	108.76936	14	15/09/2019	Larvae	Plastic container	Larvae	-	T.T.T.D
180	QN12	Binh Long district, Quan	15.279138	108.76936	14	15/09/2019	Larvae	Plastic container	Larvae	-	T.T.T.D
181	QN13	Binh Long district, Quan	15.279138	108.76936	14	15/09/2019	Larvae	Plastic container	Larvae	-	T.T.T.D
182	QN14	Binh Long district, Quan	15.279138	108.76936	14	15/09/2019	Larvae	Plastic container	Larvae	-	T.T.T.D
183	GL01	Pleiku city, Gia Lai prov	13.990133	107.992312	779	23/10/2019	Larvae	Tire	Larvae	-	C.V.D. and V.V.N
184	GL02	Pleiku city, Gia Lai prov	13.995536	107.990624	752	23/10/2019	Larvae	Tire	Larvae	-	C.V.D. and V.V.N
185	GL03	Pleiku city, Gia Lai prov	14.000466	107.988671	739	23/10/2019	Larvae	Tire	Larvae	-	C.V.D. and V.V.N
186	GL04	Pleiku city, Gia Lai prov	13.980908	107.995115	775	23/10/2019	Adult	Resident garden	Adult	Female	C.V.D. and V.V.N
187	GL05	Pleiku city, Gia Lai prov	13.979273	107.991778	787	23/10/2019	Adult	Resident garden	Adult	Female	C.V.D. and V.V.N
188	GL06	Pleiku city, Gia Lai prov	13.975484	107.992634	802	23/10/2019	Adult	Park	Larvae	-	C.V.D. and V.V.N
189	GL07	Pleiku city, Gia Lai prov	13.968628	107.998037	772	23/10/2019	Adult	Park	Larvae	-	C.V.D. and V.V.N
190	GL08	Pleiku city, Gia Lai prov	13.979668	107.985839	784	22/10/2019	Larvae	Tire	Larvae	-	C.V.D. and V.V.N
191	GL09	Pleiku city, Gia Lai prov	13.979783	107.986923	790	22/10/2019	Larvae	Tire	Larvae	-	C.V.D. and V.V.N
192	GL10	Pleiku city, Gia Lai prov	13.985519	108.002952	761	22/10/2019	Larvae	Tire	Larvae	-	C.V.D. and V.V.N
193	GL11	Pleiku city, Gia Lai prov	14.007112	107.987628	736	22/10/2019	Adult	Resident garden	Adult	Female	C.V.D. and V.V.N
194	GL12	Pleiku city, Gia Lai prov	14.008788	107.987365	749	22/10/2019	Adult	Resident garden	Adult	Female	C.V.D. and V.V.N
195	GL13	Pleiku city, Gia Lai prov	14.012437	107.988626	757	22/10/2019	Adult	Coconut	Adult	Female	C.V.D. and V.V.N
196	GL14	Pleiku city, Gia Lai prov	14.010725	107.984978	751	22/10/2019	Adult	Resident garden	Adult	Female	C.V.D. and V.V.N
197	DL01	Buon Ma Thuot city, Dal	12.67239	108.042828	469	21/10/2019	Larvae	Tire	Larvae	-	C.V.D. and V.V.N
198	DL02	Buon Ma Thuot city, Dal	12.671328	108.044881	457	21/10/2019	Larvae	Tire	Larvae	-	C.V.D. and V.V.N
199	DL03	Buon Ma Thuot city, Dal	12.680503	108.047107	481	21/10/2019	Larvae	Bamboo stump	Larvae	-	C.V.D. and V.V.N
200	DL04	Buon Ma Thuot city, Dal	12.679048	108.045798	479	21/10/2019	Adult	Cement tank	Adult	Female	C.V.D. and V.V.N
201	DL05	Buon Ma Thuot city, Dal	12.672475	108.042031	472	21/10/2019	Adult	Cement tank	Adult	Female	C.V.D. and V.V.N
202	DL06	Buon Ma Thuot city, Dal	12.669131	108.039934	459	21/10/2019	Larvae	Plastic container	Larvae	-	C.V.D. and V.V.N
203	DL07	Buon Ma Thuot city, Dal	12.677907	108.045015	477	21/10/2019	Adult	Park	Adult	Male	C.V.D. and V.V.N
204	DL08	Buon Ma Thuot city, Dal	12.67539	108.042698	474	21/10/2019	Adult	Park	Adult	Male	C.V.D. and V.V.N
205	DL09	Buon Ma Thuot city, Dal	12.662981	108.040414	439	20/10/2019	Larvae	Bamboo stump	Larvae	-	C.V.D. and V.V.N
206	DL10	Buon Ma Thuot city, Dal	12.645857	108.015589	407	20/10/2019	Adult	Tire	Adult	Female	C.V.D. and V.V.N
207	DL11	Buon Ma Thuot city, Dal	12.701003	108.060796	526	20/10/2019	Adult	Coconut	Adult	Female	C.V.D. and V.V.N
208	DL12	Buon Ma Thuot city, Dal	12.705089	108.054209	525	20/10/2019	Adult	Coconut	Adult	Female	C.V.D. and V.V.N
209	DL13	Buon Ma Thuot city, Dal	12.705759	108.052061	513	20/10/2019	Adult	Resident garden	Adult	Female	C.V.D. and V.V.N
210	DL14	Buon Ma Thuot city, Dal	12.701837	108.047881	485	20/10/2019	Adult	Resident garden	Adult	Female	C.V.D. and V.V.N
211	HCM01	Binh Chanh district, Ho C	10.724403	106.65572	-3	19/10/2019	Larvae	Plastic container	Larvae	-	C.V.D. and V.V.N
212	HCM02	Binh Chanh district, Ho C	10.72084	106.655634	2	19/10/2019	Larvae	Cement tank	Larvae	-	C.V.D. and V.V.N
213	HCM03	Binh Chanh district, Ho C	10.714937	106.656331	3	19/10/2019	Larvae	Flower pot	Larvae	-	C.V.D. and V.V.N
214	HCM04	Binh Chanh district, Ho C	10.710089	106.660897	3	19/10/2019	Adult	Tire	Adult	Male	C.V.D. and V.V.N
215	HCM05	Binh Chanh district, Ho C	10.710819	106.65515	2	19/10/2019	Adult	Coconut	Adult	Female	C.V.D. and V.V.N

216	HCM06	4 district, Ho Chi Minh c	10.754719	106.701183	4	18/10/2019	Adult	Discarded trash pot	Adult	Female	C.V.D. and V.V.N
217	HCM07	4 district, Ho Chi Minh c	10.755805	106.697407	4	18/10/2019	Adult	Tire	Adult	Female	C.V.D. and V.V.N
218	HCM08	4 district, Ho Chi Minh c	10.75746	106.696881	1	18/10/2019	Larvae	Bonsai bucket	Larvae	-	C.V.D. and V.V.N
219	HCM09	4 district, Ho Chi Minh c	10.758335	106.698008	2	18/10/2019	Larvae	Flower pot	Larvae	-	C.V.D. and V.V.N
220	HCM10	4 district, Ho Chi Minh c	10.758335	106.698008	2	18/10/2019	Adult	Resident garden	Adult	Female	C.V.D. and V.V.N
221	HCM11	7 district, Ho Chi Minh c	10.750395	106.700591	1	18/10/2019	Adult	Play ground - discarded raincoat	Adult	Female	C.V.D. and V.V.N
222	HCM12	7 district, Ho Chi Minh c	10.748245	106.697552	-1	18/10/2019	Adult	Park-plastic container	Adult	Male	C.V.D. and V.V.N
223	HCM13	7 district, Ho Chi Minh c	10.748985	106.700973	2	18/10/2019	Adult	Park-tire	Adult	Male	C.V.D. and V.V.N
224	CT01	Ninh Kieu district, Can T	10.005611	105.741194	1	17/10/2019	Larvae	Cement tank	Larvae	-	C.V.D. and V.V.N
225	CT02	Ninh Kieu district, Can T	10.005611	105.741194	1	17/10/2019	Larvae	Tire	Larvae	-	C.V.D. and V.V.N
226	CT03	Ninh Kieu district, Can T	10.002615	105.743341	1	17/10/2019	Larvae	Tire	Larvae	-	C.V.D. and V.V.N
227	CT04	Ninh Kieu district, Can T	9.999435	105.741721	2	17/10/2019	Larvae	Tire	Larvae	-	C.V.D. and V.V.N
228	CT05	Ninh Kieu district, Can T	10.001654	105.739414	2	17/10/2019	Larvae	Plastic container	Larvae	-	C.V.D. and V.V.N
229	CT06	Ninh Kieu district, Can T	9.997776	105.741442	1	17/10/2019	Larvae	Coconut	Larvae	-	C.V.D. and V.V.N
230	CT07	Ninh Kieu district, Can T	10.01008	105.753812	2	17/10/2019	Adult	Park	Adult	Female	C.V.D. and V.V.N
231	CT08	Ninh Kieu district, Can T	10.011459	105.749912	5	16/10/2019	Adult	Tire	Adult	Female	C.V.D. and V.V.N
232	CT09	Ninh Kieu district, Can T	10.010439	105.745733	5	16/10/2019	Adult	Cement tank	Adult	Female	C.V.D. and V.V.N
233	CT10	Ninh Kieu district, Can T	10.012774	105.746184	2	16/10/2019	Adult	Cement tank	Adult	Female	C.V.D. and V.V.N
234	CT11	Cai Rang district, Can Th	10.001365	105.747727	4	16/10/2019	Adult	Coconut	Adult	Female	C.V.D. and V.V.N
235	CT12	Cai Rang district, Can Th	10.000442	105.747387	6	16/10/2019	Adult	Resident garden	Adult	Male	C.V.D. and V.V.N
236	CT13	Cai Rang district, Can Th	10.002992	105.746973	3	16/10/2019	Adult	Resident garden	Adult	Male	C.V.D. and V.V.N

*C.V.D: Cuong Van Duong
V.V.N: Vinh Van Nguyen
H.V.H.: Hieu Viet Ho
T.T.T.D.: Thao Thi Thanh Dang
