# Supplementary material for: Genetic Diversity and Population Structure of the Asian Tiger Mosquito (Aedes albopictus) in Vietnam: Evidence for Genetic Differentiation by Climate Region
Source: Genes (Basel). 2021 Oct 6;12(10):1579. doi: 10.3390/genes12101579 (PMC8535633; doi:10.3390/genes12101579)
Supplement: Supplementary file 1 [file genes-12-01579-s001.zip › Supp Table S2 Haplotype survey.pdf]

Table S2. Survey of haplotype similarities between Vietnam and worldwide haplotypes.

References: Ze-Ze et al. 2020; Lee et al. 2020; Adilah-Amrannudin et al. 2018; Battaglia et al. 2016; Zhong et al. 2013; Lapadula et al. 2020; Futami et al. 2015; Unpublish

Haplotype	Climatic region																						
	Temperate														Subtropical/ Tropical								
	USA	Italy	Portugal	Greece	Canada	Spain	Russia	Austria	Albania	Turkey	China	Japan	South Korea	Taiwan	Malaysia	Thailand	India	Singapore	Panama	Brazil	Costa Rica	Colombia	Democratic Republic of the Congo
H5 (192)	KC690945, MF622084, KC690898, KC690912, KC690914, KC690932, KC690950, KC690952, KC690953, KC690955, MF185679, MF185677, MF185675, MF622084	KC690898, KC690912, KC690932, KC690935, KX383921, KX383923, KX383929, KX383934	MK995303-MK995318, MN513352-MN513359, MN513361, MF990905	MN005054, MN005055, KX383933, KX383916	MF185667, MF185668, MF185675	MT192977; KU319443	MH185667-MH817490, MT711529	MN103394	KX383930, KX383931	MK713991; MK714006, MK714008, MK714009	KY971592, KY971593, KX383929-KX383934, KX383923, KX383921, KC690898, KC690899, KC690901, KC690904, KC690910, KX266638, KX266639, KX266642, KX266643, KX266645, KX266647, KX266649, KX26649, KX266653, KX266658, KX266660, KX266664-KX266667, KX266669, KX266677, KX266690, KX266693, KX266694, KX266700, KX266709, KX266714, KX266719, KX266720, KX266723, KX266725, KX266726, KU738431, KU738430, KU738408-23, KU738406, KU738386-404, KX383929-34, KR068634	KC690898, KC690912	MG871392	KC690898, KC690914, KC690916, KC690918				KC690921	AB907801	MK575475	AB907797		
H15 (19)	KC690942, KC690896										KY971602, KY971594-KY971600, KY971591, KX266718, KX266646, KU738407, KC690942, KC690908, KC690907, KC690906, C690906	AB738090											
H17 (1)															KY982336								
H18 (1)											KC690909												
H27 (5)					MF185672						KY971601, KC690897		MG871394, MG871393										
H30 (4)	KC690917										KC690900			KC690915, KC690917									
H36 (10		KC690938																					
H1 (14)	KC690940		MK995332								JQ235749, MT890465				KY982359, MF148292, MF148287, MF148270, MF148263, MF148254		KJ410335, KJ410333			KX383924			
H3 (37)	KC690941, KC690951			KX383927							KU738424-29, MT906349-53				KY982334, KY982342, KY982353, KY982354, KY982354, KY982357, MF148290, MF148251-53, MF148258, MF148259, MF148260, MF148267, MF148279, MF148281, MF148282, MF148290	KX383925, KX383926, KX383928							MN299017
H13 (1)																						MN997607	
H16 (1)															KY982336								
H40 (2)															MF148289, KY982356								
H43 (3)															MF148284, MF148278, KY982366								
H50 (1)															MF148261								
H59 (3)															MF148283, MF148276, KY982364								
