# Supplementary material for: Genetic Diversity and Population Structure of the Asian Tiger Mosquito (Aedes albopictus) in Vietnam: Evidence for Genetic Differentiation by Climate Region
Source: Genes (Basel). 2021 Oct 6;12(10):1579. doi: 10.3390/genes12101579 (PMC8535633; doi:10.3390/genes12101579)
Supplement: Supplementary file 1 [file genes-12-01579-s001.zip › Suppl Tables S3, S4, S5.pdf]

Table S3. Correlations between 19 bioclimatic variables in Vietnam.

	bio1	bio2	bio3	bio4	bio5	bio6	bio7	bio8	bio9	bio10	bio11	bio12	bio13	bio14	bio15	bio16	bio17	bio18	bio19
**bio1**	1.00																		
**bio2**	-0.42	1.00																	
**bio3**	0.45	0.53	1.00																
**bio4**	-0.64	-0.22	-0.93	1.00															
**bio5**	0.86	-0.62	0.01	-0.21	1.00														
**bio6**	0.97	-0.34	0.58	-0.78	0.74	1.00													
**bio7**	-0.81	0.10	-0.78	0.94	-0.41	-0.92	1.00												
**bio8**	0.36	-0.61	-0.42	0.36	0.63	0.16	0.16	1.00											
**bio9**	0.93	-0.19	0.69	-0.85	0.64	0.98	-0.94	0.01	1.00										
**bio10**	0.72	-0.76	-0.26	0.07	0.94	0.56	-0.20	0.78	0.44	1.00									
**bio11**	0.93	-0.16	0.73	-0.88	0.63	0.98	-0.95	0.05	0.98	0.41	1.00								
**bio12**	0.20	0.09	0.27	-0.34	0.09	0.28	-0.32	-0.31	0.28	-0.04	0.29	1.00							
**bio13**	0.03	0.03	-0.02	-0.05	0.04	0.07	-0.07	-0.35	0.09	0.01	0.04	0.84	1.00						
**bio14**	0.13	-0.58	-0.67	0.53	0.52	-0.01	0.33	0.38	-0.10	0.65	-0.18	0.02	0.32	1.00					
**bio15**	-0.26	0.27	0.00	0.06	-0.37	-0.23	0.09	-0.43	-0.13	-0.31	-0.20	0.56	0.77	-0.04	1.00				
**bio16**	-0.05	0.13	0.05	-0.08	-0.10	0.01	-0.07	-0.39	0.04	-0.15	0.01	0.91	0.95	0.11	0.82	1.00			
**bio17**	0.03	-0.54	-0.69	0.57	0.42	-0.09	0.37	0.32	-0.17	0.56	-0.26	0.04	0.36	0.98	0.03	0.16	1.00		
**bio18**	-0.78	0.35	-0.41	0.62	-0.62	-0.81	0.73	0.00	-0.83	-0.47	-0.78	0.05	0.11	-0.16	0.39	0.25	-0.06	1.00	
**bio19**	0.66	-0.37	0.10	-0.26	0.65	0.61	-0.44	0.07	0.64	0.61	0.52	0.28	0.42	0.57	0.12	0.27	0.54	-0.66	1.00

Abbreviation: **bio1-**Annual Mean Temperature; **bio2**-Mean Diurnal Range (Mean of monthly (max temp - min temp)); **bio3-**Isothermality (BIO2/BIO7) (×100); **bio4**-Temperature Seasonality (standard deviation ×100); **bio5**-Max Temperature of Warmest Month; **bio6**-Min Temperature of Coldest Month; **bio7-**Temperature Annual Range (BIO5-BIO6); **bio8-**Mean Temperature of Wettest Quarter; **bio9**-Mean Temperature of Driest Quarter; **bio10**-Mean Temperature of Warmest Quarter; **bio11**-Mean Temperature of Coldest Quarter; **bio12**-Annual Precipitation; **bio13**-Precipitation of Wettest Month; **bio14**-Precipitation of Driest Month; **bio15**-Precipitation Seasonality (Coefficient of Variation); **bio16**-Precipitation of Wettest Quarter; **bio17**- Precipitation of Driest Quarter; **bio18**-Precipitation of Warmest Quarter; **bio19**-Precipitation of Coldest Quarter.

Correlation >0.8 between variables are indicated in yellow.

Table S4. Pairwise genetic (F_{ST}, below the diagonal) and geographic distances (km, above the diagonal) for *Aedes albopictus* populations in Vietnam.

	**S1**	**S2**	**S3**	**S4**	**S5**	**S6**	**S7**	**S8**	**S9**	**S10**	**S11**	**S12**	**S13**	**S14**	**S15**	**S16**
**S1**	0	117.2	129.9	164.1	288.8	345.6	283.6	375.3	380.1	428.2	859.5	967.7	1049	1184	1339	1398
**S2**	0.0421	0	94.6	256.8	362.2	390.9	290.1	386.2	350.7	376.3	805.9	914	980.2	1110	1250	1302
**S3**	**0.0609**	0.0747	0	202.8	285.6	300.9	195.5	291.7	264.3	302.2	734.6	842.9	920.3	1055	1210	1271
**S4**	**0.28898**	**0.3194**	**0.1361**	0	131.3	213	217.3	282.9	342.6	416	823.3	928.7	1033	1175	1355	1428
**S5**	**0.31401**	**0.3579**	**0.1841**	-0.0207	0	105.6	190.5	209.6	317.7	396.8	762.3	863.3	982.4	1128	1326	1409
**S6**	**0.30704**	**0.3350**	**0.1561**	-0.0333	-0.0038	0	137.8	113.6	234.1	321.1	660.5	760.1	883.1	1029	1233	1321
**S7**	0.01961	**0.0983**	0.0487	**0.3221**	**0.3621**	**0.3550**	0	96.23	127.5	208.5	606.2	712	815.8	958.6	1145	1224
**S8**	0.01816	0.0313	0.0144	**0.1573**	**0.18297**	**0.1610**	0.0209	0	129.6	214.5	553.2	655.3	772.9	918.3	1120	1207
**S9**	0.04217	0.0337	**0.0690**	**0.2443**	**0.2845**	**0.2530**	0.0312	0.0000	0	86.97	485.3	592.5	690.1	832.1	1018	1098
**S10**	**0.0622**	0.0420	**0.1492**	**0.3883**	**0.4337**	**0.4216**	0.0569	0.0400	-0.0090	0	432.4	540.7	623.8	726.6	939.2	1016
**S11**	0.00223	0.0237	**0.0967**	**0.3845**	**0.4222**	**0.4140**	0.0218	0.0427	0.0256	0.0311	0	108.4	230	376	609.6	721.3
**S12**	0.0281	0.0273	**0.1276**	**0.3597**	**0.4039**	**0.3877**	**0.0963**	**0.0418**	0.0092	0.0026	0.0191	0	165.9	300.3	550.7	672.1
**S13**	-0.0021	**0.0927**	**0.0738**	**0.3544**	**0.3927**	**0.3899**	0.0194	0.0535	**0.0886**	**0.1130**	0.0172	**0.0712**	0	146.6	386.1	506.2
**S14**	0.00269	**0.0863**	**0.1203**	**0.3740**	**0.4080**	**0.4021**	0.0606	**0.0754**	**0.0717**	**0.0911**	0.0133	0.0418	-0.0103	0	258.5	388.4
**S15**	0.01612	0.0362	**0.1306**	**0.3900**	**0.4346**	**0.4240**	0.0774	0.0430	0.0109	0.0290	-0.0108	-0.0190	0.0405	0.0092	0	134
**S16**	**0.07342**	0.0346	**0.1647**	**0.3896**	**0.4319**	**0.4198**	**0.1481**	**0.0636**	**0.0430**	0.0345	**0.0593**	0.0110	**0.1409**	**0.1044**	0.0143	0

Significant values of pairwise F_{ST} are indicated in bold

Table S5. Genetic differentiation of *Aedes albopictus* populations in Vietnam.

No.	Studied region	MtDNA gene	No. of sequences	Length (bp)	No. of haplotype	Haplotype diversity	Nucleotide diversity	References
**Native regions**								
**1**	**Vietnam**	***COI***	**236**	**658**	**65**	**0.904**	**0.00313**	Current study
**2**	China	*COI*	119	700	25	0.663	0.0052	Ruiling, Xuejun and Zhong, 2018
**3**	China	*COI*	128	518	55	0.946	0.0161	Zhang et al., 2017
**4**	Pacific-Ocean	*COI*	1044	445	52	0.83	0.0037	Maynard et al., 2017
**5**	Laos	*COI*	155	1337	44	0.416 - 0.942	0.0004-0.0023	Motoki et al., 2019
**6**	Korea	*COI*	292	1485	38	0.111-0.806	0.0003-0.0015	Lee et al., 2019
**7**	Thailand	*COI*	55	518	5	0.626	0.0538	Zhang et al., 2017
**8**	Singapore	*COI*	10	518	4	0.8	0.0028	Zhang et al., 2017
**9**	Japan	*COI*	24	518	3	0.606	0.0014	Zhang et al., 2017
**Invasive regions**								
**10**	Mexico	Cytb	125	317	25	0.492 ± 0.05	0.003 ± 0.006	Pech-May et al., 2016
**11**	Cameroon	COI	153	550	4	0.24	0.0010	Kamgang et al., 2011
		ND5	153	400	4	0.24	0.0010	
**12**	Congo	COI	127	638	3	0.24	0.0001	Kamgang et al., 2018
**13**	East-Adriatic	COI	39	464	4	0.282	0.0001	Žitko et al., 2011
		ND5	39	393	1	0	0	
**14**	Madagascar	COI	77	518	3	0.414	0.0009	Zhang et al., 2017
**15**	America							Zhong et al. 2013
	Los - Angeles 2011	COI	34	1433	6	0.5160 ±0.09	0.0014	
	New - Jersey	COI	30	1433	5	0.5460 ±0.10	0.0007	
	Texas	COI	31	1433	9	0.7260 ±0.08	0.0015	
	Hawaii	COI	32	1433	8	0.6960 ±0.07	0.0015	
