# Supplementary figures and images for: Genetic Diversity and Population Structure of the Asian Tiger Mosquito (Aedes albopictus) in Vietnam: Evidence for Genetic Differentiation by Climate Region
Source: Genes (Basel). 2021 Oct 6;12(10):1579. doi: 10.3390/genes12101579 (PMC8535633; doi:10.3390/genes12101579)

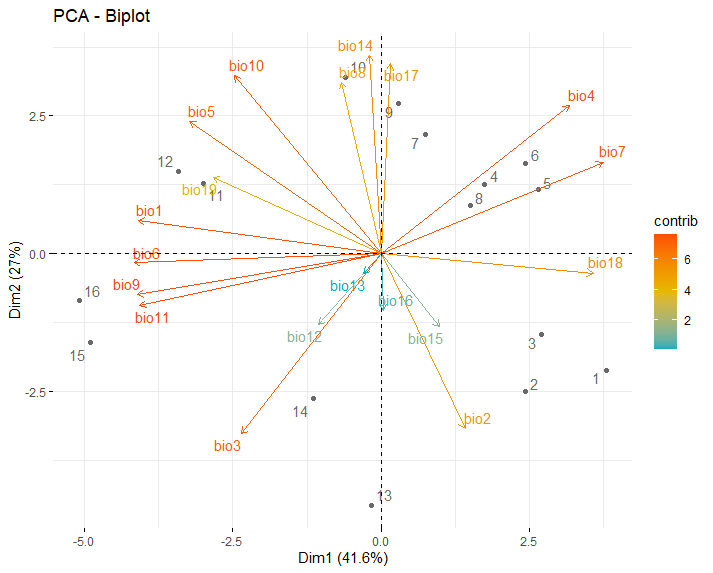

Supplement: Supplementary file 1 [file genes-12-01579-s001.zip › Figure S1. PCA of 19 bio variables.png]

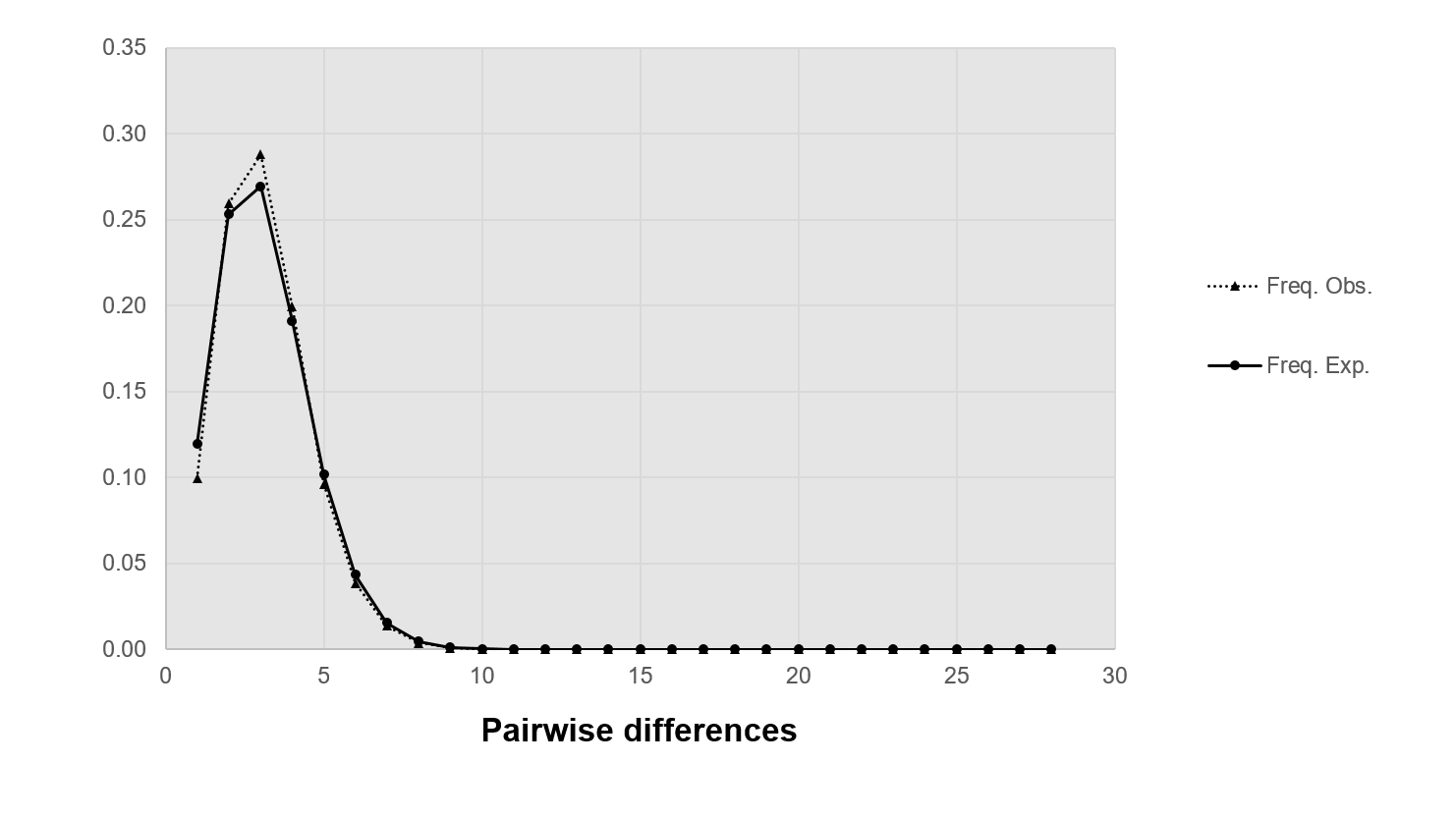

Supplement: Supplementary file 1 [file genes-12-01579-s001.zip › Figure S2. Mismatch distribution analysis.tif]

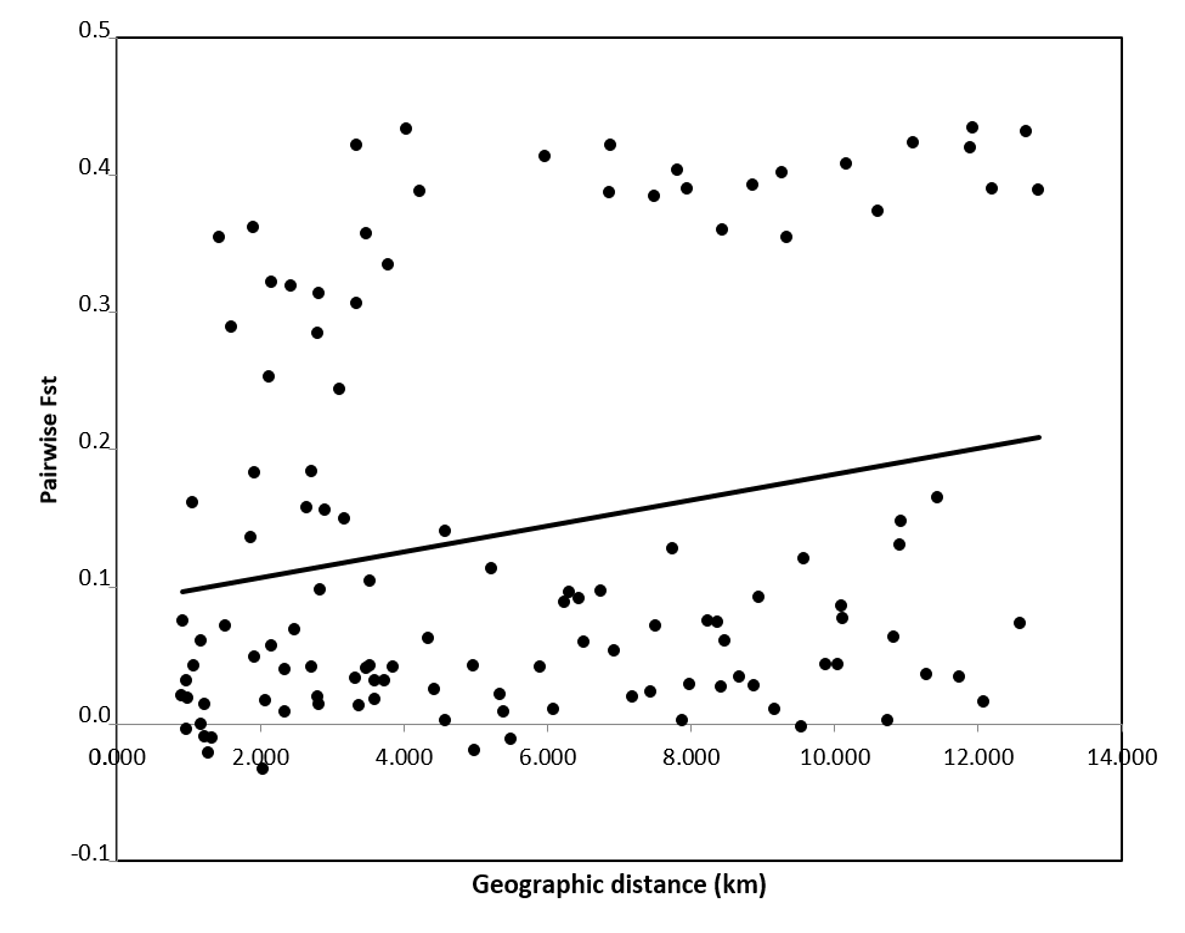

Supplement: Supplementary file 1 [file genes-12-01579-s001.zip › Figure S3. Plot of isolation by distance.tif]
